# The role of retinoid-related orphan receptor-α in cigarette smoke-induced autophagic response

**DOI:** 10.1186/s12931-022-02034-5

**Published:** 2022-05-04

**Authors:** Hak-Su Kim, Chang Hyeok An, Danielle Teller, Su-Jin Moon, Gi Won Hwang, Jin Woo Song

**Affiliations:** 1 Veterans Health Service Medical Center, Veterans Medical Research Institute, Seoul, Republic of Korea; 2 Department of Pulmonary and Critical Care Medicine, Asan Medical Center, University of Ulsan College of Medicine, 88, Olympic-Ro 43-Gil, Songpa-Gu, Seoul, 05505 Republic of Korea; 3 Division of Pulmonary and Critical Care Medicine, Brigham and Women’s Hospital, 75 Francis Street, Boston, MA USA; 4 Division of Pulmonology, Department of Internal Medicine, Hanil General Hospital, Seoul, Republic of Korea

**Keywords:** Retinoid-related orphan receptor-α, Chronic obstructive pulmonary disease, Autophagy, p53, Damage-regulated autophagy modulator

## Abstract

**Background:**

Retinoid-related orphan receptor-α (RORα) and autophagy dysregulation are involved in the pathophysiology of chronic obstructive pulmonary disease (COPD), but little is known regarding their association. We investigated the role of RORα in COPD-related autophagy.

**Methods:**

The lung tissues and cells from a mouse model were analyzed for autophagy markers by using western blot analysis and transmission electron microscopy.

**Results:**

Cigarette smoke increased the LC3-II level and decreased the p62 level in whole lung homogenates of a chronic cigarette smoking mouse model. Although cigarette smoke did not affect the levels of p62 in Staggerer mutant mice (RORα^sg/sg^), the baseline expression levels of p62 were significantly higher than those in wild type (WT) mice. Autophagy was induced by cigarette smoke extract (CSE) in Beas-2B cells and in primary fibroblasts from WT mice. In contrast, fibroblasts from RORα^sg/sg^ mice failed to show CSE-induced autophagy and exhibited fewer autophagosomes, lower LC3-II levels, and higher p62 levels than fibroblasts from WT mice. Damage-regulated autophagy modulator (DRAM), a p53-induced modulator of autophagy, was expressed at significantly lower levels in the fibroblasts from RORα^sg/sg^ mice than in those from WT mice. DRAM knockdown using siRNA in Beas-2B cells inhibited CSE-induced autophagy and cell death. Furthermore, RORα co-immunoprecipitated with p53 and the interaction increased p53 reporter gene activity.

**Conclusions:**

Our findings suggest that RORα promotes autophagy and contributes to COPD pathogenesis via regulation of the RORα-p53-DRAM pathway.

**Supplementary Information:**

The online version contains supplementary material available at 10.1186/s12931-022-02034-5.

## Background

Chronic obstructive pulmonary disease (COPD) is the third leading cause of death worldwide [1] and is characterized by an incompletely reversible airflow obstruction associated with respiratory symptoms, such as cough, excessive sputum production, and dyspnea [2]. COPD mortality is expected to double by the end of the next decade [3, 4]. More than 75% of COPD diagnoses are associated with cigarette smoking, which is known to play a role in COPD pathogenesis [2, 5]; however, only a minority of smokers develop the disease, suggesting that other factors, including genetic susceptibility, are involved. Many investigations on the key pathophysiological mechanisms have focused on varied cellular processes—from dysregulated protease activity to epithelial cell apoptosis.

The role of retinoid-related orphan receptor-α (RORα) in the pathogenesis of COPD has been previously reported [6]; the RORα protein expression level is higher in patients with COPD and in epithelial cells and fibroblasts exposed to cigarette smoke extract (CSE). This upregulation of RORα is due to the induction of DNA damage by cigarette smoke and RORα plays a crucial role in cell death. In the absence of RORα, CSE-induced apoptosis is suppressed [6]. Thus, it has been suggested that RORα is important in promoting emphysema through epithelial cell apoptosis.

The understanding of the role of autophagy, which is another fundamental cellular process, in various lung diseases, including COPD, is rapidly expanding [7, 8]. Autophagy is a basic, evolutionarily conserved process that serves a homeostatic function, triggering a cellular stress response [9]. In certain conditions, it can also promote cell death. This latter function has been found to be prominent in the pathogenesis of COPD in which autophagy is induced by cigarette smoke and leads to epithelial cell death, thus, playing a key role in alveolar destruction and emphysema development [10, 11].

The relationship between p53 and RORα [12] and the known, but complex, relationship between p53 and autophagy [13] led us to investigate whether RORα regulates autophagy in COPD. Considering autophagy is a cell-dependent process [14], we investigated the modulation of autophagy by RORα in both lung epithelial cells and lung fibroblasts. In this study, we provide evidence that RORα can regulate autophagy both under basal conditions and in response to cigarette smoke. We demonstrate that, in the absence of RORα, the normal autophagic response to cigarette smoke is absent. We also demonstrate that p53 and DRAM, which are known regulators of autophagy [15], are mediators of the effect of RORα on autophagy.

## Methods

### Cell culture

Beas-2B cells and A549 cells, human lung epithelial cells, and p53-deficient H1299 lung cancer cells were obtained from the American Type Culture Collection (ATCC, Manassas, VA, USA) and maintained in DMEM (Thermo Fisher, Waltham, MA, USA) containing 10% fetal bovine serum (FBS, HyClone, Logan, UT, USA) and antibiotics (Invitrogen, Carlsbad, CA, USA). The cells were seeded into six-well plates, and incubated for 24 h. At 70–80% confluence, cells were exposed to the indicated concentration of CSE with 10% FBS for the indicated time.

To prepare CSE, Kentucky 1R3F research-reference filtered cigarettes (The Tobacco Research Institute, University of Kentucky, Lexington, KY, USA) were smoked using a peristaltic pump. The cigarette filter was removed prior to the experiments. The smoke of four cigarettes was bubbled through 40 mL of cell growth medium, and this solution was regarded as 100% strength CSE. CSE was frozen immediately in liquid nitrogen and stored at − 80 °C until use.

For the isolation of primary fibroblasts, mouse lung tissues were cut into 1 × 1 mm^2^ pieces and cultured in DMEM containing 10% FBS and antibiotics at 37 °C for 7–10 days with medium changes every 3 days. Cells in passages 2–5 were used for all experiments. Cell viability was determined using the 3-(4,5-dimethylthiazol-2-yl)-2,5-diphenyl tetrazolium bromide assay.

### Animals

All experimental procedures were conducted in accordance with the Guiding Principles for the Care and Use of Animals, and protocols were approved by the Animal Care and Handling Committee of the Asan Medical Center (2015-13-125, Seoul, South Korea), and 6-week-old C57BL/6J mice were obtained from Orient Bio (Seongnam, South Korea) and acclimated for 1 week before the experiments. Staggerer mutant (sg) and wild type (WT) mice were obtained from Jackson Laboratories (Bar Harbor, ME, USA). These mice were exposed to cigarette smoke using a smoking chamber for 5 days/week for 6 months, as described previously [10]. They were then euthanized and their lungs were harvested, snap frozen in liquid nitrogen, and stored at − 80 °C.

### Transmission electron microscopy (TEM)

Primary lung fibroblasts from RORα^WT^ and RORα^sg/sg^ mice were treated with 10% CSE and fixed with ice-cold 2.5% glutaraldehyde for 30 min. After washing in PBS, the cells were post-fixed in 1% osmium tetroxide and embedded in Epon. Thin sections were stained using uranyl acetate/lead citrate and viewed via TEM. For unbiased quantification, five randomly selected fields were used, and the number of autophagosomes per 10 µm^2^ in each field was counted manually.

### Immunoblotting

To obtain total protein lysates, the cells or lung tissues were homogenized using radioimmunoprecipitation assay buffer (1% NP40, 0.5% sodium deoxycholate, 0.1% sodium dodecyl sulfate, 1 mM ethylene diamine tetraacetic acid, 1 mM ethylene glycol tetraacetic acid, 1 mM Na_3_VO_4_, 20 mM NaF, 0.5 mM dl-dithiothreitol, 1 mM phenylmethane-sulfonyl fluoride, and a protease inhibitor cocktail in PBS; pH 7.4) and centrifuged at 14,000×*g* and 4 °C for 15 min. The total protein content of the supernatant was quantified using a BCA protein assay kit (Pierce, Rockford, IL, USA). Protein extracts (10–30 µg) were separated using 4–20% SDS-polyacrylamide gel electrophoresis and transferred to polyvinylidene difluoride membranes in a transfer buffer containing 25 mM Tris–HCl, 192 mM glycine, and 10% methanol. The membranes were blocked with 5% bovine serum albumin in Tris-buffered saline with 0.1% Tween 20 and incubated with specific primary antibodies followed by incubation with secondary antibodies. Immunoreactive bands were visualized using an enhanced chemiluminescence detection system and analyzed using image analysis software Quantity One (Bio-Rad Laboratories, Hercules, CA, USA). Antibodies against RORα, DRAM, α-actinin, and β-actin were purchased from Santa Cruz Biotechnology (Santa Cruz, CA, USA), and antibodies against LC3, p62, and p53 were obtained from Cell Signaling Technology (Boston, MA, USA). Antibodies against autophagy-related gene (Atg) 5-Atg12 protein were also purchased from Abcam (Cambridge, UK).

### Plasmid and siRNA transfection

H1299 cells were transfected with the GFP-p53 plasmid using the PolyFect Transfection Reagent (Qiagen, Valencia, CA, USA) according to the manufacturer's instructions. RORα (sc-38862), DRAM (sc-96209), and control siRNAs (sc-36869) were purchased from Santa Cruz Biotechnology. Fluorescein-conjugated control siRNA was used as an indicator of the transfection efficiency as a negative control. siRNA transfection was performed according to the manufacturer’s instructions.

### Statistical analysis

Data are expressed as mean ± standard error from at least three independent experiments and were analyzed using GraphPad Prism 5 software (La Jolla, CA, USA). One-way analysis of variance was used followed by a Newman–Keuls multiple comparison test for more than three groups or an unpaired *t*-test for two groups. All *p*-values were two-tailed with the statistical significance set at *p* < 0.05.

## Results

### Cigarette smoke upregulates RORα expression and induces autophagy in a mouse model

**Fig. 1:**
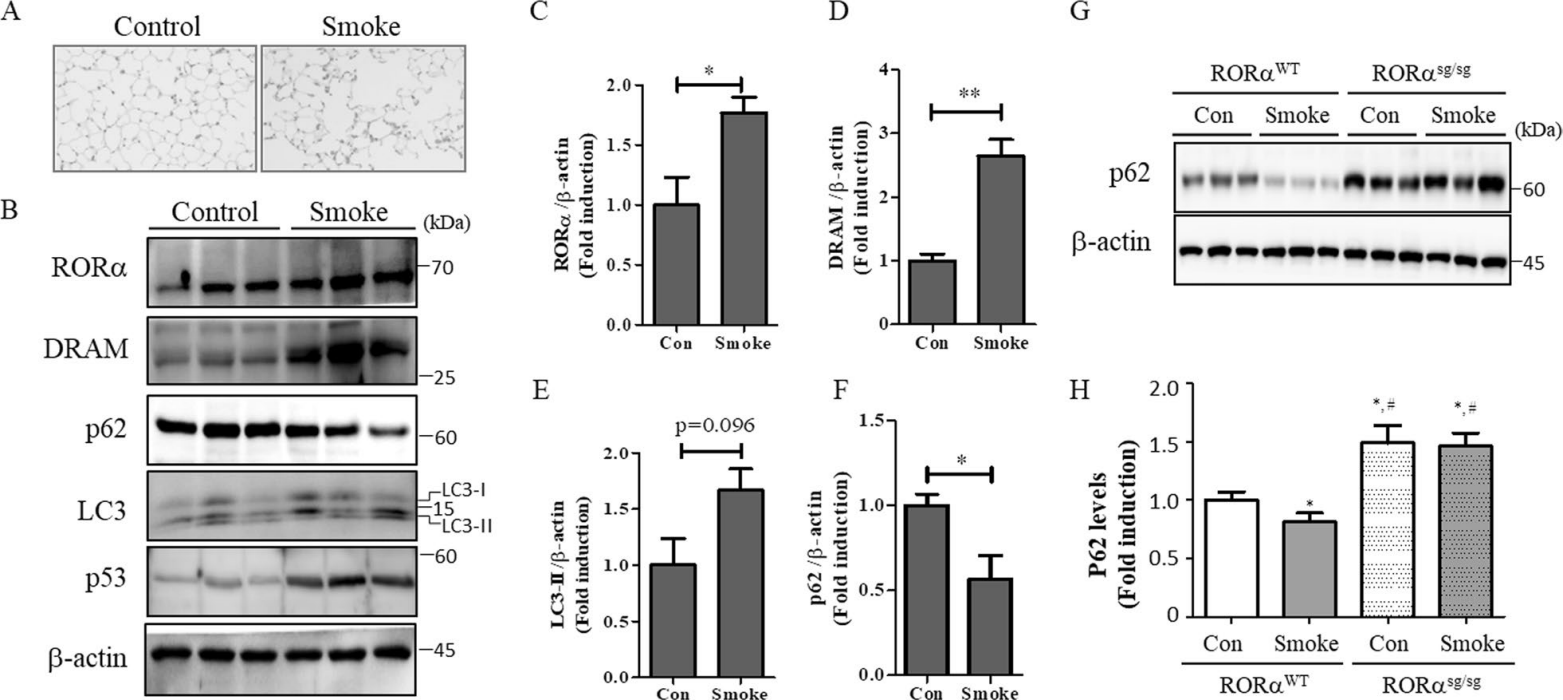
RORα regulates CSE-induced autophagy in a mouse model. **A** Mice were exposed to smoke for 5 days/week for 24 weeks. Three mice were assigned to each group. The histopathologic features of lungs are shown by H&E stain. **B** Homogenates of mouse lung tissues were subjected to western blot assays using anti-RORα, anti-DRAM, anti-p62, anti-LC3, anti-p53, and anti-β-actin antibodies. Densitometry was used to analyze the fold changes of the levels of **C** RORα, **D** DRAM, **E** LC3B, and **F** p62. * and ** indicate p < 0.05 and p < 0.01, respectively. **G** RORα^WT^ and RORα^sg/sg^ mice were exposed to smoke for 5 days/week for 24 weeks. Homogenates of mouse lung tissues were subjected to western blot assays using anti-p62 and anti-β-actin antibodies. Representative blots of proteins in the lung tissues are shown. **H** Densitometry was used to analyze the fold changes of the levels of p62 for four groups of mice: RORα^WT^ + Con group (n = 4), RORα^WT^ + Smoke group (n = 10), RORα^sg/sg^ + Con group (n = 4) and RORα^sg/sg^ + Smoke group (n = 7). *p < 0.05 compared with the RORα^WT^ mice of the nonsmoking (RORα^WT^ + Con) group, and ^#^p < 0.01 compared with the RORα^WT^ mice of the smoking (RORα^WT^ + Smoke) group

The inhibition of RORα can protect against emphysema [6] and regulates autophagy flux [16]. Autophagy is activated in the lung tissues of patients with COPD and epithelial cells in response to CSE; there is evidence that autophagy contributes to cell death [10]. To understand the relationship between RORα and autophagy, we demonstrated the expression pattern of autophagy-related proteins (damage-regulated autophagy modulator [DRAM], LC3-II, and p62) and RORα in an experimental smoke exposure mouse model. Mice were exposed to smoke for 5 days/week for 24 weeks. As shown in Fig. 1A, mice exposed to smoke exhibited distended alveoli and enlarged air sacs compared to the relatively well-formed alveolar units in control mice. The expression levels of RORα were significantly higher in the lung tissues of cigarette smoke exposure mice than in those of control mice (Fig. 1B, C). Notably, the expression levels of autophagy regulators DRAM (p = 0.004) and LC3-II (p = 0.09) were higher and those of p62 (p = 0.04) were lower in the lung tissues of cigarette smoke exposure mice than in those of control mice (Fig. 1B, D–F).

Subsequently, to investigate the role of RORα in cigarette smoke-induced autophagy, we used sg mice, which are well-characterized by the functional loss of RORα and lack an encoding part of the ligand binding domain of RORα (RORα^sg/sg^). We exposed RORα^sg/sg^ and control mice (RORα^WT^) to cigarette smoke for 5 days/week for 6 months and assessed the p62 levels in the whole-lung homogenates. The baseline expression levels of p62 in RORα^sg/sg^ mice were significantly higher than those in WT mice (Fig. 1G, H). However, the cigarette smoke did not affect the levels of p62 in the RORα^sg/sg^ mice. The expression levels of p62 in the smoking RORα^WT^ mice were significantly lower than those in non-smoking RORα^WT^ mice, as was expected from the increasing autophagy and activation of RORα (Fig. 1G, H). Similar results were also found in experiments using other autophagic markers (Atg5–Atg12) (Additional file 1: Fig. S1).

### CSE induces autophagy via the expression of RORα in epithelial cells

**Fig. 2:**
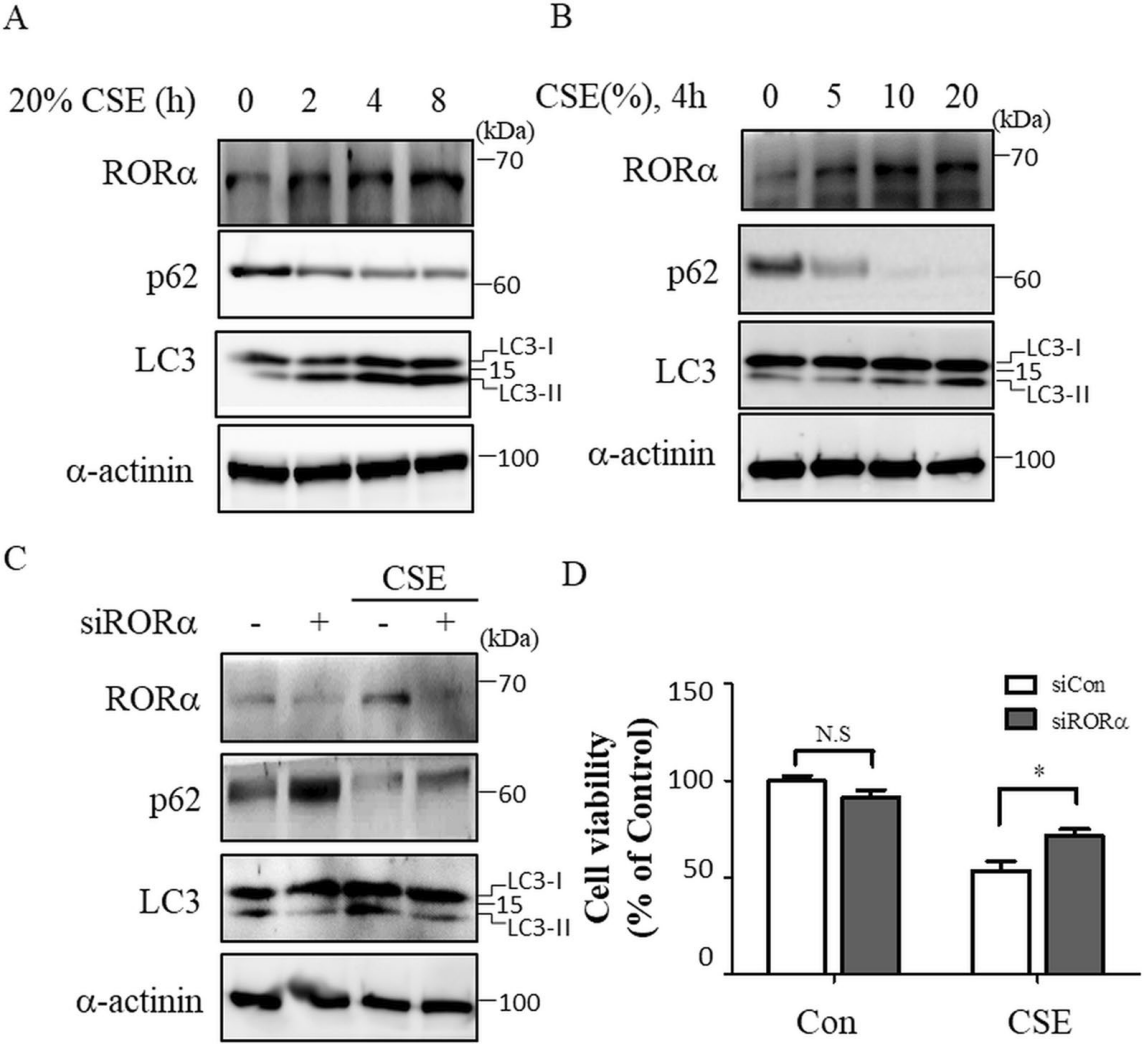
Effect of RORα on CSE-induced autophagy in Beas-2B cells. **A** Beas-2B cells were exposed to 20% CSE for the indicated time. **B** Cells were exposed to the indicated concentration of CSE for 4 h. Under these conditions, the total cell extracts were subjected to western blot assays using anti-RORα, anti-p62, anti-LC3, and anti-α-actinin antibodies. **C** Beas-2B cells were transfected with the control or RORα siRNAs for 48 h. The cell lysates were prepared after treatment with 20% CSE for 24 h and subjected to western blot. **D** Under the same conditions as in **C**, the cells were analyzed for their viability using an MTT assay. The data are expressed as the mean ± S.E. of 2 independent experiments with quadruplicate determinations. *p < 0.01

We first investigated the association between the expression of RORα and autophagy induced by CSE in human lung epithelial cells. CSE induced the expression of RORα in Beas-2B cells (Fig. 2A, B) and A549 cells (Additional file 1: Fig. S1). CSE also increased the expression level of LC3-II and reduced the expression levels of p62 in Beas-2B cells (Fig. 2A, B) and A549 cells (Additional file 1: Fig. S2). The effects of RORα knockdown using specific siRNA on CSE-induced autophagy and cell death were investigated to verify the function of RORα. Beas-2B cells were transfected with RORα-specific or control siRNA for 48 h and treated with CSE. The knockdown of RORα slightly increased the basal level of p62, rescued the CSE-induced reduction in p62 levels, and suppressed the CSE-induced increase in LC3-II levels (Fig. 2C). Cell viability assays also demonstrated that the knockdown of RORα significantly abated CSE-induced cell death but did not influence basal cell viability (Fig. 2D). These results suggested that CSE-induced RORα expression increases autophagy in Beas-2B cells.

### CSE-induced autophagy is impaired in the fibroblasts from RORα^sg/sg^ mice

**Fig. 3:**
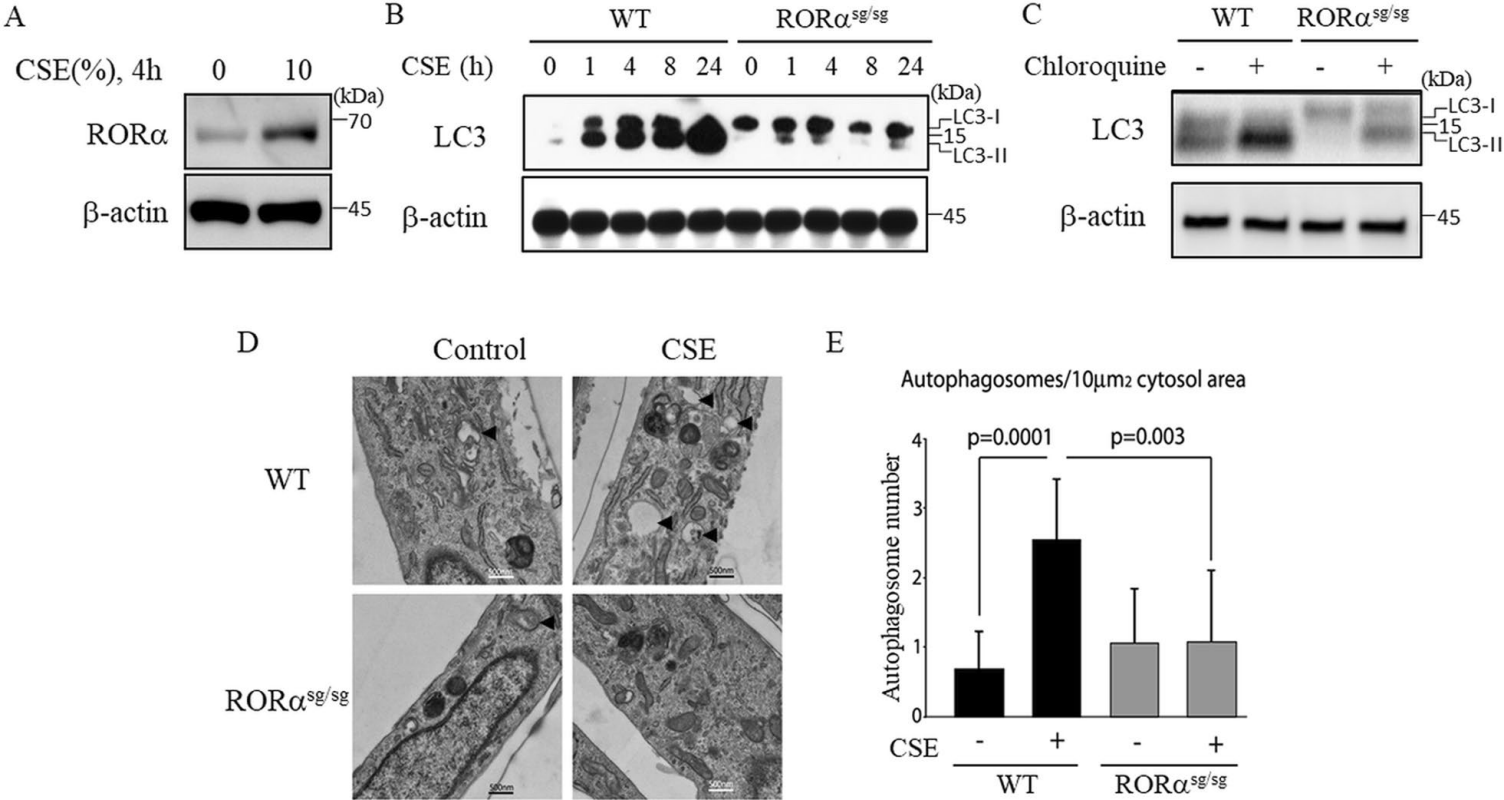
CSE-induced autophagy is impaired in the fibroblasts from RORα^sg/sg^ mice. **A** Primary lung fibroblasts isolated from RORα^WT^ treated with 10% CSE for 4 h. **B** Primary lung fibroblasts were isolated from RORα^WT^ and RORα^sg/sg^ mice and treated with 10% CSE for the indicated time. **C** Autophagic flux in response to 10% CSE treatment was assessed based on the LC3 turnover using chloroquine in the fibroblasts from RORα^WT^ and RORα^sg/sg^ mice. **D** Transmission electron microscopy of the fibroblasts from RORα^WT^ and RORα^sg/sg^ mice treated with 10% CSE. Arrowheads indicate autophagosomes. **E** The number of autophagosomes per 10 µm^2^ is shown

To confirm the function of RORα in CSE-induced autophagy, we next investigated the effects of CSE in primary lung fibroblasts isolated from RORα^WT^ and RORα^sg/sg^ mice. Primary lung fibroblasts isolated from RORα^WT^ mice treated with CSE increased the expression levels of RORα (Fig. 3A) and LC3-II (Fig. 3B); in contrast, the primary lung fibroblasts isolated from RORα^sg/sg^ mice treated with CSE demonstrated little accumulation of LC3-II (Fig. 3B). To complement the static measurement of the LC3 protein levels, we investigated the autophagic flux in RORα^sg/sg^ cells as a response to CSE. Treating cells with chloroquine before harvesting blocked autolysosome maturation, and the LC3 turnover was measured. Compared to primary lung fibroblasts isolated from RORα^WT^ mice exposed to 10% CSE, those from RORα^sg/sg^ mice had decreased autophagic flux (Fig. 3C). Finally, we analyzed the cells treated with CSE by measuring the autophagosome number using electron microscopy (Fig. 3D). As shown in Fig. 3E, the primary lung fibroblasts isolated from RORα^WT^ mice had an expected increase in the autophagosome number upon exposure to CSE, but RORα^sg/sg^ cells showed no significant increase above the baseline levels. Taken together, these results demonstrate that, in the absence of RORα, cigarette smoke-induced autophagy is inhibited in primary lung fibroblasts.

### CSE-induced expression of DRAM involves RORα activation

**Fig. 4:**
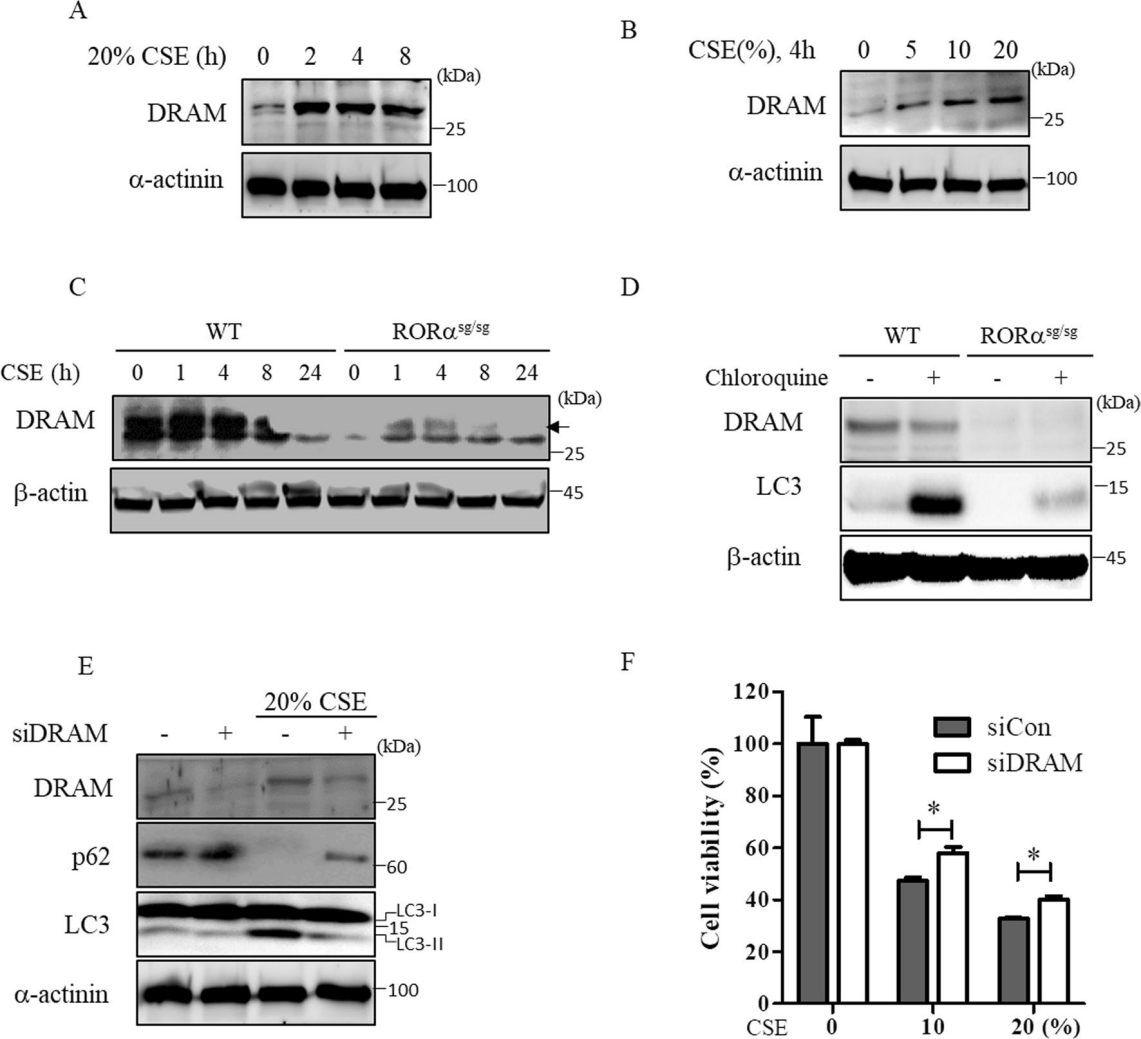
Effect of DRAM on RORα-dependent autophagy. **A** Beas-2B cells were exposed to 20% CSE for the indicated time. **B** Cells were exposed to the indicated concentrations of CSE for 4 h. Under these conditions, the total cell extracts were subjected to western blot using anti-DRAM and anti-α-actinin antibodies. **C** Primary lung fibroblasts were isolated from RORα^WT^ and RORα^sg/sg^ mice and treated with 20% CSE for the indicated time. **D** CSE-treated primary fibroblasts were exposed to 100 µM chloroquine for 4 h. **E** Beas-2B cells were transfected with control or DRAM siRNAs for 48 h. The cell lysates were prepared after treatment with 20% CSE for 4 h and subjected to western blot. **F** Beas-2B cells were transfected with the control or RORα siRNAs for 48 h and exposed to the indicated concentration of CSE. Cells were analyzed for their viability using an MTT assay. The data are expressed as the mean ± S.E. of 2 independent experiments with triplicate determinations

DRAM is involved in autophagy activation by p53 [15]. We next investigated the role of DRAM in RORα-mediated autophagy induction. We found that CSE induced the expression of DRAM in human lung epithelial cells (Fig. 4A, B and Additional file 1: Fig. S1) and that the levels of DRAM were significantly decreased in fibroblasts isolated from RORα^sg/sg^ mice compared to those in fibroblasts isolated from RORα^WT^ mice (Fig. 4C). Meanwhile, chloroquine did not change the levels of DRAM in RORα^sg/sg^ fibroblasts but did decrease the levels of DRAM in CSE-treated primary fibroblasts (Fig. 4D). To elucidate whether DRAM is necessary for RORα to modulate autophagy, we subsequently silenced DRAM in Beas-2B cells. The knockdown of DRAM rescued the CSE-induced reduction in p62 levels and suppressed the CSE-induced increase in LC3-II levels (Fig. 4E). Cell viability assays also demonstrated that the knockdown of DRAM significantly rescued CSE-induced cell death (Fig. 4F).

### Effect of RORα on CSE-induced autophagy is achieved in a p53-dependent manner

**Fig. 5:**
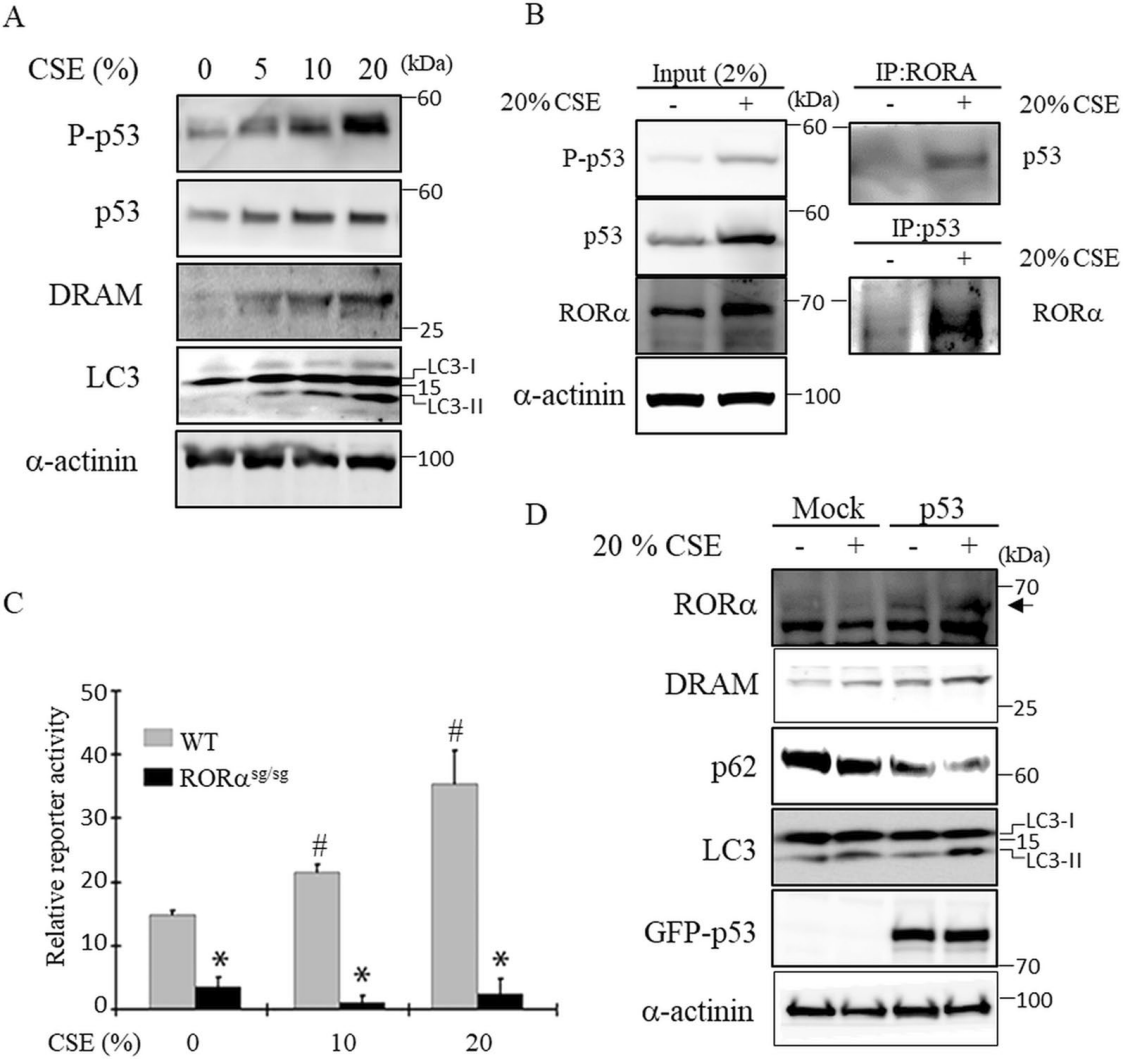
RORα directly interacts with p53 and increases p53 transcriptional activity. **A** Beas-2B cells were exposed to the indicated concentration of CSE for 16 h. **B** Co-immunoprecipitation of RORα and p53 at the endogenous level in Beas-2B cells treated with CSE for 4 h. **C** A p53 reporter assay was performed on the lung fibroblasts from RORα^WT^ and RORα^sg/sg^ mice treated with the indicated concentration of CSE. *p < 0.05 compared with 0% CSE treatment in WT fibroblasts, and ^#^p < 0.05 compared with WT fibroblasts. **D** H1299 cells were transfected with a mock or p53 WT plasmid for 24 h and exposed to 20% CSE for 4 h. Total cell extracts were subjected to western blot using anti-RORα, anti-DRAM, anti-p62, anti-LC3, and anti-α-actinin antibodies

p53 has been extensively studied as a regulator of autophagy, primarily in cancer biology. p53 has the ability to both induce and inhibit autophagy in a context-dependent manner [13]. Based on a previous study on RORα, we investigated the relationship between RORα and p53 [12]. Because DRAM is a regulator of p53-activated autophagy, we attempted to further understand the relationship between RORα and p53. CSE induced the expression of phospho-p53, p53, and DRAM in Beas-2B cells in a dose-dependent manner (Fig. 5A). Beas-2B cells were treated with CSE for 4 h and co-immunoprecipitation of RORα and p53 was performed using the cell lysate. The results showed that CSE treatment increased the interaction between RORα and p53 (Fig. 5B). We also used a p53 reporter assay to assess p53 activity in response to CSE in lung fibroblasts. Consequently, CSE increased the p53 transcriptional activity in lung fibroblasts from WT mice in a dose-dependent manner (Fig. 5C). In contrast, it did not affect the p53 transcriptional activity in the lung fibroblasts from RORα^sg/sg^ mice. To further verify the function of p53, we investigated the CSE-induced autophagy in H1299 cells, which are p53-mutant lung epithelial cells. H1299 cells were transfected with an expression vector encoding GFP-WT p53. The overexpression of p53^WT^ in H1299 cells increased the levels of RORα and DRAM; their increased levels were accelerated by CSE and p53^WT^, ultimately promoting CSE-induced autophagy (Fig. 5D). These results indicate that the autophagy-enhancing effects of RORα after CSE treatment might be primarily mediated by p53.

## Discussion

**Fig. 6:**
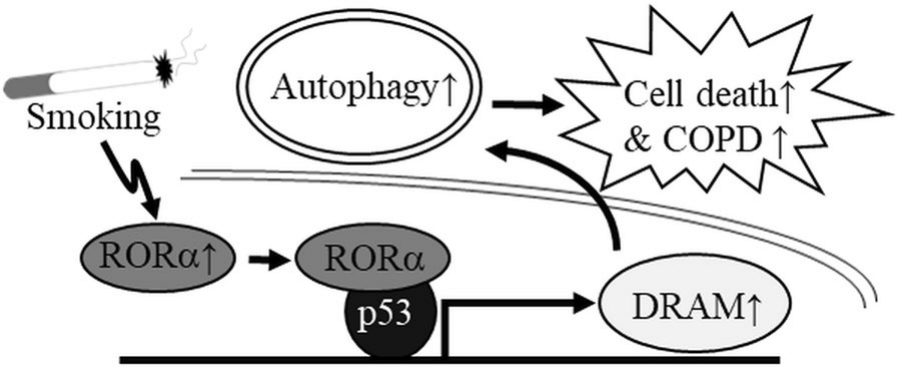
Schematic summary showing the proposed role of RORα on cigarette smoke-induced autophagy. The increase in RORα by smoking induces activity of the p53-DRAM axis through direct binding with p53 and then induces an increase in autophagy, leading to an increase in cell death

In this study, we identified the important relationship between the fundamental cellular process of autophagy and the nuclear receptor RORα in COPD pathogenesis. Our results demonstrate that RORα is an effective autophagy regulator in in vitro and in vivo COPD models. In addition, the p53-DRAM axis, which is activated by RORα, is important for cigarette smoke-induced autophagy and cell death (Fig. 6).

Autophagy plays an important role in chronic lung disease, including COPD [7, 8], pulmonary fibrosis [17, 18], cystic fibrosis [19], and lung injury [20]. Numerous studies have shown that cigarette smoke increases autophagy and promotes epithelial cell death in vitro and in vivo [8, 10, 11]. In the lung tissues of patients with COPD, the expression level of LC3-II, Atg4, and Atg7 is increased; CSE increases autophagy by upregulating LC3-II expression [8]. LC3 knockdown also decreases CSE-induced apoptosis in lung epithelial cells and decreases emphysema occurrence [8]. However, low doses of CSE (0.5–1%) have shown that the inhibition of autophagy increases CSE-induced cell senescence or ROS production [21, 22]. Accordingly, the role of autophagy in the pathogenesis of COPD may be dependent on the degree of exposure to cigarette smoke. In low concentrations or low stress conditions, cigarette smoke-induced autophagy may be beneficial by reducing cell senescence and ROS production. However, exposure to high concentrations beyond the normal range for autophagy result in excessive activation of autophagy, leading to epithelial cell dysfunction and death. A previous study also reported the role of RORα in DNA damage as it is related to smoking-related lung diseases [6]. In the present study, cigarette smoke-induced RORα expression increased the autophagic response via the p53-DRAM pathway. Therefore, we hypothesized that inhibition of RORα activity decreased autophagy and was a negative regulator of epithelial cell death by cigarette smoke.

DNA damage induces cell cycle arrest, and abnormal regulation of the cell cycle often leads to the development of many diseases, such as cancer and COPD [23–25]. Some studies have begun focusing on the relationship between cell cycle and COPD [24, 25]. For example, the cell cycle gene *p53* is associated with COPD development and progression [6]. Tumor suppressor p53, which is a key regulator of cell death, is upregulated in DNA damage conditions and determines the cellular fate [26]. Recently, the role of p53 as an autophagy regulator has been studied [26]. *DRAM*, a p53 target gene, is an inducer of autophagy-mediated apoptosis [15]. Moreover, the levels of p53 are higher in the lungs of patients with COPD compared to those in control lungs, which is consistent with increased apoptosis [6, 27]. *RORα* is a direct p53 target gene containing the p53 response element in its promoter and is also an important regulator that exerts its role by increasing the apoptosis level in DNA damage conditions [12, 28]. In the present study, the involvement of a previously unknown pathway, the RORα-p53-DRAM pathway, in cigarette smoke-induced autophagy was demonstrated using a chronic cigarette smoke exposure mouse model. CSE increased the levels of RORα, p53, and DRAM, and the inhibition of RORα activity by RORα^sg/sg^ or siRNA decreased CSE-induced autophagy. CSE-induced RORα directly binds to p53 then increases p53 transcriptional activity. Furthermore, the functional loss of p53 resulted in the failure of RORα induction and cell death in response to CSE, indicating that CSE-induced RORα expression occurs in a p53-dependent manner.

Epidemiological data indicate that disruption of the circadian rhythmicity is associated with the pathogenesis of COPD [29]. COPD symptoms worsen in the morning. A study has indicated that patients who experience morning symptoms have a high risk of exacerbation [30]. The circadian rhythm in the forced expiratory volume in 1 s in stable COPD peaks at 4:00 pm and dips at around 4:00 am [31]. RORα is one of the nuclear receptor superfamily elements that plays a critical role in the regulation of the circadian clock [32, 33]. RORα expression is induced in response to various cellular stresses [6, 34]. Additionally, RORα is expressed at high levels in the lung tissues of patients with COPD [6]. In our study, CSE increased RORα expression levels, leading to an increased p53 function and autophagy and subsequently inducing cell death. The functional inhibition of RORα also decreased the CSE-induced pathological effects, including autophagy and epithelial cell death in the lung. Our results suggest that the functional inhibition of RORα is useful for the reduction of pathological autophagy and that RORα is a good target for developing COPD treatments.

Our study had some limitations. First, we used only one model among the different approaches to imitate COPD in animal models. These models include exposing mice to cigarette smoke, lipopolysaccharides, or elastase as well as genetic modifications [35]. The exposure protocol also varied, such as whole body or nose-only exposure to cigarette smoke. In the present study, we used whole body exposure of mice to cigarette smoke. Second, the effect of RORα at low concentrations of CSE was not investigated although the high and low concentrations of CSE had different effects on autophagy. Finally, there was no evaluation of the effect of RORα inhibition on other physiological phenomena. The risk of RORα inhibition, such as carcinogenesis, aging, and host defense, must be taken into account for the development of therapeutics. Therefore, further studies are required to obtain a comprehensive understanding of the RORα function in the pathogenesis of COPD.

## Conclusions

In conclusion, we used in vitro and in vivo models of COPD to investigate the significance of cigarette smoke-induced RORα in p53-DRAM-induced autophagy and confirmed that cigarette smoke-dependent autophagy and cell death are promoted when RORα and p53 are co-expressed in lung epithelial cells. These results indicate that RORα is a novel inducer of autophagy in cigarette smoke exposure conditions and that the functional inhibition of RORα can protect against the development of COPD. Furthermore, these findings improve our understanding of COPD pathophysiology.

## Supplementary Information

**Additional file 1: Figure S1.** Autophagy markers, Atg5–Atg12 are reduced in RORα^sg/sg^ mice. **Figure S2.** CSE upregulates RORα expression and induces autophagy in A549 cells.

## Data Availability

The datasets used and/or analyzed during the current study are available from the corresponding author on reasonable request.

## Abbreviations

RORα: Retinoid-related orphan receptor-α
COPD: Chronic obstructive pulmonary disease
CSE: Cigarette smoke extract
sg: Staggerer mutant
WT: Wild type
DRAM: Damage-regulated autophagy modulator
TEM: Transmission electron microscopy
