# Supplementary material for: The role of retinoid-related orphan receptor-α in cigarette smoke-induced autophagic response
Source: Respir Res. 2022 May 4;23:110. doi: 10.1186/s12931-022-02034-5 (PMC9066967; doi:10.1186/s12931-022-02034-5)

Additional File 1

**The role of retinoid-related orphan receptor-α in cigarette smoke-induced autophagic response**

Hak-Su Kim^1,2^, [Chang Hyeok An](https://www.ncbi.nlm.nih.gov/pubmed/?term=An%20CH%5BAuthor%5D&cauthor=true&cauthor_uid=22744720)^3,4^, [Danielle Teller](https://www.ncbi.nlm.nih.gov/pubmed/?term=Morse%20D%5BAuthor%5D&cauthor=true&cauthor_uid=22744720)^3^, Su-Jin Moon^2^, Gi Won Hwang^2^, Jin Woo Song^2^**^*^**

^1^Veterans Medical Research Institute, Veterans Health Service Medical Center, Seoul, Republic of Korea

^2^Department of Pulmonary and Critical Care Medicine, Asan Medical Center, University of Ulsan College of Medicine, Seoul, Republic of Korea

^3^Division of Pulmonary and Critical Care Medicine, Brigham and Women's Hospital, 75 Francis Street, Boston, MA, USA

^4^Division of Pulmonology, Department of Internal Medicine, Hanil General Hospital, Seoul, Republic of Korea

Fig. S1. Autophagy markers, Atg5-Atg12 are reduced in RORα^sg/sg^ mice

RORα^WT^ and RORα^sg/sg^ mice were exposed to smoke for 5 days/week for 24 weeks. Homogenates of mouse lung tissues were subjected to western blot assays using anti-Atg5-Atg12 and anti-β-actin antibodies. Representative blots of proteins in the lung tissues are shown. Densitometry was used to analyze the fold changes of the levels of Atg5-Atg12 for four groups of mice: RORα WT + Con group (n = 4), RORα WT + Smoke group (n = 10), RORα sg/sg + Con group (n = 4) and RORα sg/sg + Smoke group (n = 7). * p < 0.05 compared with the RORα WT mice of the nonsmoking (RORα WT + Con) group.


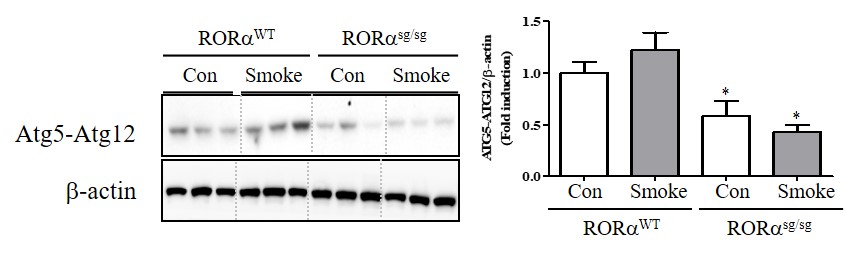


Fig. S2. CSE upregulates RORα expression and induces autophagy in A549 cells.

A549 cells were exposed to the indicated concentration of CSE for 4 h. Total cell extracts were subjected to Western blot assays using anti-RORα, anti-p53, anti-DRAM, anti-p62, anti-LC3, and anti-α-actinin antibodies.


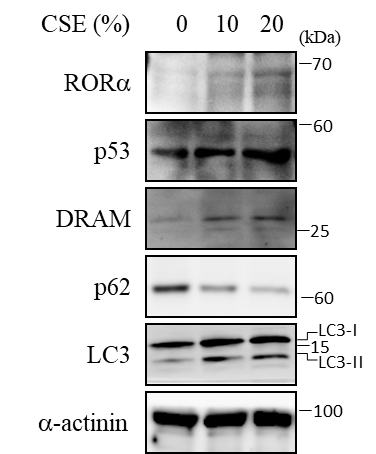

Supplement: Supplementary file 1 — Additional file 1: Figure S1. Autophagy markers, Atg5–Atg12 are reduced in RORαsg/sg mice. Figure S2. CSE upregulates RORα expression and induces autophagy in A549 cells. [file 12931_2022_2034_MOESM1_ESM.docx]
